# The Eleventh Annual Meeting of the American Medical Association

**Published:** 1858-06

**Authors:** 


*The Eleventh Annual Meeting of the American Medical Association.*—The American Medical Association held its eleventh annual meeting at Washington, commencing the first Tuesday in May. From the admirable location of the meeting, and its contiguity to the larges tcities of the Union, it was expected that this would be one of the most brilliant and interesting that had ever been held since its organization; and in this we have not been disappointed: the President of the United States, General Cass, Senator Douglas, as well as many other distinguished personages, both of our profession and among the laymen, entertained the members of the association with munificent and cordial hospitality. Among other expeditions, the members had an excursion to Mount Vernon, which was variegated by a plank shad-bake, with the "et ceteras." We were unfortunately unable to be present at the meeting, but the city of Buffalo was admirably represented, by a delegation consisting of Profs. Hamilton, White and Flint, and Drs. Wilcox and Lockwood, the latter gentleman giving additional lustre to the party by virtue of his office of Mayor of the city.

We will give our readers an abstract of the proceedings, which we compile from the Washington daily papers.

First Day.—The Association met in the lecture room of the Smithsonian Institute, and was called to order at a quarter past eleven o'clock, A. M., by Dr. Condie, of Philadelphia, when the chair was taken by the president, Dr. Paul F. Eve, of Nashville, Tennessee. Vice presidents Breckinridge of Kentucky, Reese of New York, and Campbell of Georgia, were also upon the platform; and at their table were the efficient secretaries, Drs. Foster, of Tennessee, and Semmes, of this city, to whom we are under obligations.

Rev. Byron Sunderland, D. D., at the invitation of the president, offered an eloquent and appropriate prayer, invoking the blessing of Almighty God upon the convention.

Dr. Harvey Lindsley, of this city, chairman of the committee of arrangements, then delivered an address, welcoming the Association, and offering them the hospitalities of the city; alluding in appropriate terms to the dignity of the body which was about to hold its meeting, regretting that there were no extensive hospitals, or venerable universities, but mentioning among the objects of interest which were laid open to the members, "one spectacle which can be witnessed no where else—a sight worth a pilgrimage from the remotest bounds of the republic—we can show you the home of Washington—that Mecca of the American people—to which every patriotic hear will ever turn with sentiments of devoted affection and filial reverence." (Applause.)

The Secretary then called the roll by states, as it had been made out up to the commencement of the meeting, and the following number of delegates responded: Maine 2, New Hampshire 8, Connecticut 18, Vermont 1,

Massachusetts 40, Rhode Island 5, New York 73, New Jersey 25, Pennsylvania 66, Delaware 4, Maryland 24, District of Columbia 25, Virginia 8, North Carolina 8, South Carolina 10, Georgia 12, Alabama 1, Kentucky 9, Tennessee 7, Indiana , Illinois 12, Michigan 3, Iowa 3, Missouri 4, Ohio 14, California 1, American Medical Society of Paris 1, U. S. Navy 2. (When the name of Dr. Harvey, who has come from California expressly to attend this convention, was called, there was loud applause. Other members were announced at different times during the day, and when the Association adjourned there were *four hundred and six* names registered. A large increase is expected to-day.

Dr. Lindsley, chairman of the committee of arrangements, reported that it had been decided to hold but one business session each day, from 9 A. M. until 3 P. M. He also announced that the President of the United States would be happy to receive, with those members of the Association who might call at the executive mansion at eight o'clock in the evening, such ladies as may accompany them. (Applause.)

On motion, the Association confirmed the appointment of Dr. J. M. Snyder to fill a vacancy in the committee of arrangements.

On motion, it was decided that a nominating committee of one from each state represented should be raised, the delegation of each state selecting its representative therein. A brief discussion upon the propriety of permitting the army and navy delegations to appoint separate members of this committee was decided by the president in favor of their having the privilege, and the decision was sustained by the Association.

There was then a recess of fifteen minutes, during which the different delegations assembled in various parts of the lecture room to choose their representatives in the committee. After the meeting was again called to order, the Secretary read the list as follows:

*Committee of Nomination.*—Job Holmes, Maine; George H. Hubbard, New Hampshire; P. Pineo, Vermont; Ebenezer Alden, Massachusetts; Ashbel Woodward, Connecticut; J. Mauran, Rhode Island; H. D. Berkly, New York; J. P. Colman, New Jersey; Isaac Hays, Pennsylvania; H. F. Askew, Delaware; S. P. Smith, Maryland; Noble Young, District of Columbia; A. S. Payne, Virginia; W. H. McKee, North Carolina; Wm. T. Wragg, South Carolina; Joseph P. Logan, Georgia; J. T. Hargraves, Alabama; R. J. Breckinridge, Kentucky; J. Berrian Lindsley, Tennessee; Wm. M. McPheeters, Missouri; George Mendenhall, Ohio; Calvin West, Indiana; A. H. Luce, Illinois; Zina Pitcher, Michigan; Thomas O. Edwards, Iowa; O. Harvey, California; and George Clymer, United States navy.

On motion, Drs. Bohrer, of D. C., Flint, of New York, and Hargraves, of Alabama, were appointed by the president a committee on special essays.

Dr. David M. Reese, of New York, presented and read a written apology for having recommended for a position in Blockley Hospital, Philadelphia, Dr. McClintock, who had been expelled from the Association for a violation of the ethics and the etiquette of the profession, by publishing a work on "domestic medicine," &c.

On motion of Dr. Condie, of Philadelphia, the apology was accepted, and ordered to be entered upon the minutes.

Dr. Bryan, of Philadelphia, who had also recommended Dr. McClintock, made a verbal adoption of Dr. Reese's apology, the reception of which was warmly debated. Dr. C. C. Cox, of Maryland, opposed and Dr. Condie advocated the reception. Dr. A. B. Palmer, of Michigan, moved the previous question on a motion to refer the subject to a committee, which was lost. The apology of Dr. Bryan was then accepted. (It was rumored in the hall that Dr. McClintock will be reinstated during the session of the Association.)

The president then delivered, in a clear voice, and with a pleasing oratorical effect, his annual address.

(We extract from this address some passages which are peculiarly interesting to our readers, and shall take occasion to review it more at length in the appearance of the volume of transactions.) After some general remarks, the president stated that prizes had been awarded by the Association to the authors of the following essays, viz:

On the corpus luteum of menstruation and pregnancy, for 1851.

On the variation of pitch in percussion and respiratory sounds in physical diagnosis, for 1852.

On the cell, its physiology, pathology, and philosophy.

And on the surgical treatment of certain fibrous tumors of the uterus, heretofore considered beyond the resources of art, for 1853.

On a new method of treating ununited fractures and certain deformities of the osseous system, for 1854.

On the statistics of placenta prævia, for 1855.

On the physiology and chief pathological relations of the arterial circulation, for 1856.

On the excito-secretory system of nerves, its relations to physiology and pathology.

And on experimental researches in relation to the nutritive value and physiological effects of albumen, starch, and gum, when singly and exclusively used as food, for 1857.

Carefully prepared reports have been published by the Association of the various epidemics and diseases which have prevailed during the past ten years throughout our widely extended country, and the mortuary statistics and public health of our large cities minutely ascertained. Charts, maps, diagrams, tables, and plates have been freely employed to illustrate these subjects, so important to the general welfare of the people. Every state and territory, every large city and sick community, with scarcely an exception, has had its hygienic condition explored by this body; and dysentery and cholera, typhoid and yellow fevers have specially claimed the attention of our members. The communications on deformities after fractures, found in our eighth, ninth, and tenth volumes, constitute the basis of the best monograph ever issued from the press. This work, it may be predicted, will do more than all others to check the reckless and speculative spirit of suits for mal-practice against medical men; for in addition to teaching a useful lesson to the profession in the prognosis of fractures, its testimony is so conclusive in reference to the usual results of these accidents, that judicial decisions must hereafter be regulated by it.

Besides these contributious to medical knowledge, this Association has taken action to prevent the importation into our country of "worthless, adulterated, and misnamed drugs, medicines, and chemical preparations," for which a member of the United States Senate has publicly declared that if we had accomplished nothing else, this alone should have entitled us to the gratitude of the nation; it recommended to the different states the adoption of a regular system of registration of births, marriages, and deaths; memorialized Congress to secure steerage passengers in our emigrant vessels medical attention, and due amount of space between decks; appointed a committee to ascertain the best means of preventing the introduction of disease by emigrants into our large cities; and considered many interesting individual cases.

On motion, the thanks of the Association were voted to the president for his able and instructive address, a copy of which was solicited for publication.

Dr. Grafton Tyler, of Georgetown, D. C., chairman of the committee on prize essays, reported that the essays received were three in number, each of which had been examined with great care; considering, first, the intrinsic merits of each essay, and then their merits in relation to each other. The first prize was awarded to "an essay on the clinical study of the heart sounds, in health and disease," bearing the motto: "*Clinica clinice demonstranda.*" The second prize was awarded to "an essay on vision and some of the anomalies as revealed by the opthalmiscope," bearing the motto: "*Dux hominum medicus est.*"

Dr. Tyler then proceeded to open the sealed envelopes bearing the above-named mottoes, and containing the names of the writers of the essays. The first was written by Dr. Austin Flint, of Buffalo, New York; and the second by Dr. Montrose A. Pallen, of St. Louis, Missouri. This is the second time Dr. Flint has won this distinguished honor, and the third time that it has been awarded to Buffalo since the Association was organized, eleven years ago.

On motion, the report of the committee was accepted and adopted. Doctors Flint and Pallen were then invited to give *resumês* of their essays, which they did.

Dr. Lindsley, from the committee of arrangements, then presented an invitation from Dr. Nichols to visit the Insane Asylum, and another from Rev. Mr. McGuire to visit Georgetown College.

On motion of Dr. Hamilton, of New York, these invitations were accepted, and the thanks of the Association were returned therefor.

On motion of Dr. Lindsley, the Hon. Doctors Fitch, of Indiana, Chaffee, of Massachusetts, Clawson and Robbins, of New Jersey, and Shaw, of North Carolina, members of Congress, and Dr. Peter Parker, ex-commissioner to China, were elected "members by invitation," and requested to participate in the proceedings of the Assocation.

On motion, assistant surgeon Frederick A. Rose, of the British navy, who so nobly volunteered his services on board the United States ship Susquehanna at Port Royal, and who came in her to New York, devoting himself to the sick crew, was unanimously elected a "member by invitation," and invited to take a seat upon the platform. (Applause.) It was announced that Dr. Rose has left the city.

Dr. Francis G. Smith, of Philadelphia, chairman of the committee on publication, made his report, showing the expense of publishing the annual volume.

Dr. Caspar Wistar, of Philadelphia, presented his annual report of receipts and expenditures, showing a balance on hand of $806. Accompanying the treasurer's report was a resolution providing that the back volumes on hand, when over two years old, shall be sold at two dollars a volume, and that volumes V, VII, VIII, and IX, of which there are a surplus, be sold at $5 a set to any member.

The special committee on medical education, of which Dr. G. W. Morris, of Philadelphia, is chairman, were called upon to report. There was no response; and, on motion, the subject was referred to the committee on nominations.

Dr. A. B. Palmer, chairman of the committee on medical literature, asked leave to defer his report until Wednesday, at 10 o'clock; which was granted.

A report was made by the committee on nominations, which was accepted; and the Association then elected the following officers: *President*, Dr. Harvey Lindsley, of Washington city. *Vice Presidents*, Drs. W. L. Sutton, of Kentucky; Thomas O. Edwards, of Iowa; Josiah Crosby, of New Hampshire; and W. C. Warren, of North Carolina. *Secretary*, Dr. A. J. Semmes, of Washington city. (The other secretary will be elected when the location of the next Association is selected.) *Treasurer*, Caspar Wistar, of Philadelphia.

On motion, Drs. Flint, of New York, Gross, of Pennsylvania, and Gibbes, of South Carolina, were appointed a committee to conduct the president elect to the chair.

Dr. Lindsley, having been introduced to the Association by the retiring president, Dr. Eve, made a few pertinent remarks, acknowledging the honor as the highest he had ever been called upon to receive, and the highest that any medical man in America can receive. (Applause.) Unaccustomed to preside over so large a body, and having had but little practice in presiding over smaller assemblages, he must throw himself upon the forbearance of the Association, and look to the members for support in the discharge of his official duties. (Applause.)

On motion, the thanks of the Association were voted to the retiring officers for the able and impartial manner in which they have discharged the duties of their respective offices. (Applause.)

On motion, the ex-presidents of the Association present were invited to take seats on the platform.

The committee on medical topography and epidemics was called by states. A paper from the member from Maine stated that he will report next year. There was no response from New Hampshire, Vermont, Rhode Island, Connecticut, or Massachusetts. Dr. Smith, of New Jersey, read an able report on New Jersey, and the Association then adjourned until this morning at nine o'clock.

*Evening Hospitalities.*—At eight o'clock in the evening the delegates and the ladies who have accompanied them paid a visit by invitation to the executive mansion. The east room, with the adjacent suite of drawing rooms, were brilliantly lighted, and were filled by about five hundred gentlemen, representing all sections of the country, and a hundred or more ladies. One of the delegates had seen upwards of fourscore years—others have but just entered upon the practice of their profession.

The President received his gnests, as they were successively presented by Dr. Cornelius Boyle, chairman of the committee of arrangements, with his accustomed cordiality, and afterwards moved about in the east room, engaging in conversation with the groups there gathered. The entire cabinet was present, with J. B. Henry, Esq., marshal Selden, and commissioner Blake.

From the executive mansion the delegates generally proceeded to Georgetown, where they were hospitably entertained at the residences of Dr. Grafton Tyler, at the corner of Gay and Washington streets, and of Dr. Riley, No. 91 Gay street. A cordial welcome and good cheer awaited them at the houses of each of these distinguished practitioners.

There were a large number of arrivals at the different hotels last evening, and an interesting session may be expected to-day.

---

SECOND DAY.—The Association was called to order by the president, Dr. Harvey Lindsley, and A. J. Semmes, one of the secretaries, read the minutes of the first day's proceedings; which were adopted.

On motion of Dr. Watson, of New York, Dr. Delafield, of New York, one of the first officers of the Association, was invited to take a seat on the platform.

On motion of Dr. Atkinson, of Virginia, an amendment to the constitution was received, providing that no person shall be recognised as a member, or admitted as a delegate at meetings of the Association who has been expelled from any state or local medical association until relieved action of such state or local ässociation. (Applause.)

Dr. Atkinson supported the adoption of this amendment in an eloquent speech, contending that the admission of any gentleman who has been rebuked by any state or local association, and is under its ban, is a rebuke to that association. He urged the acceptance of the amendment, and trusted that until the constitution be so amended it shall be the rule of action.

Dr. Bond, of Maryland, asked to have the qualifications requisite for a seat read. He desired information as to the ethical qualifications for membership.

Dr. Watson, of New York, stated that, as by the constitution it was necessary to have amendments lie over one year, this was not a question for present debate.

The president decided that debate was not in order, and the amendment was accordingly laid on the table for consideration at the next annual meeting.

Dr. Boyle, chairman of the committee of arrangements, proposed the names of Doctors Huff and Knight, who were elected "members by invitation."

Dr. A. B. Palmer, of Michigan, chairman of the committee on medical literature, made an able and interesting report.

On motion, the report was accepted, and ordered to be published.

On motion, Dr. Bozeman, of Alabama, was elected a "member by invitation."

*Report on Medical Education.*—Dr. James R. Wood, chairman of a special committee on medical education, made a lengthy report, discussing: 1st, primary medical schools; 2d, the number of professorships in medical colleges; 3d, the length and number of terms during the year; 4th, the requisite qualifications for graduation; 5th, such other subjects of a general character as to give uniformity to our medical system.

On motion, the report was accepted and referred to the committee on publication, the accompanying resolution being laid on the table.

The committee on nominations reported Louisville, Ky., as the place of meeting in 1859, and nominated Dr. S. S. Bemis, of that city, as second secretary. They also nominated the following standing committees:

*Committee on Publication*—Dr. Gurney Smith, Pa., chairman; Drs. Caspar Wistar, Pa.; A. J. Semmes, D. C.; S. M. Bemis, Ky.; S. L. Hollinsworth, Pa.; S. Lewis, Pa.; H. F. Askew, Del.

*Committee on Medical Literature*—Dr. John Watson, N. Y., chairman; Drs. L. A. Smith, N. J.; C. G. Comegys, Ohio; R. W. Gibbs, S. C.; W. M. McPheeten, Mo.

*Committee on Prize Essays*—Dr. J. B. Flint, N. Y., chairman; Drs. M. Goldsmith, N. J.; H. Miller, Ky.; Calvin West, Ind.

*Committee on Medical Education*—Dr. G. W. Norris, Pa., chairman; Drs. A. H. Luce, Ill.; E. R. Henderson, S. C.; G. R. Grant, Tenn.; T. S. Powell, Ga.

*Committee of Arrangements*—R. J. Breckinridge, Ky., chairman; Drs. G. W. Ronald, B. M. Wible, D. W. Goodall, D. D. Thompson, N. B. Marshall, G. W. Burglass, R. C. Hewett, and A. B. Cook, all of Kentucky.

The report was accepted, the nominations were confirmed, and the committee received permission to sit again.

On motion of Dr. Hamilton, of Buffalo, the resolutions attached to the report of the committee on medical education were taken from the table.

Dr. Watson moved the appointment of a committee to consider the resolutions and report to-morrow (this) morning.

Dr. Bond thought that the subject had already been sufficiently discussed. It had been brought up year after year, occupying much of the time of the Association, and he trusted that it would receive immediate consideration.

Dr. Davis, of Illinois, wished to have the subject made a special order for some time prior to the adjournment of the convention.

Dr. Rogers, of New York, wished to have the report printed, that all might have an opportunity of examining lt and the propositions which it imbodies.

Dr. Wood defended his report as a conservative report, just alike to the profession and to the laymen. He did not believe that any good could arise from a further discussion of the subject. None had arisen in years past—none could arise now. It was a bill of conciliation and of adjustment. Laymen of the profession merited censure for sending men to college not qualified for the profession, and colleges merited censure for sending men out not qualified to practise the healing art. (Applause.) He approved of the motion of Dr. Watson, that the report be submitted to a committee of delegates from colleges.

A debate on a call for the previous question on Dr. Watson's resolution hen ensued, in which several gentlemen joined, each one apparently having a different idea of "parliamentary law," and neither of them displaying a very correct knowledge of the subject. It was remarked by an old member of the Association that "parliamentary discussion must be a *local epidemic*."

The report was finally referred to a select committee, to be composed of one member from each delegation representing a medical college or school.

On motion, thanks were voted to the late secretary, Dr. Foster, and his successor, Dr. Bemis, took his seat.

Dr. Hamm, of Pennsylvania, moved a suspension of the rules for the purpose of reconsidering the resolution of Dr. Condie, accepting the apology tendered by Dr. Bryan. The vote upon suspending the rules stood—ayes 111, noes 82. The president ruled that a two-third vote was necessary, and decided the question as lost.

An appeal was taken from the decision of the chair, and the decision was not sustained. A vote was then taken, and the resolution accepting the apology of Dr. Bryan was reconsidered by a vote of—yeas 142, nays 70.

An attempt was then made to connect the resolution with that accepting the apology of Dr. Reese, but it was decided that it would first be necessary to dispose of the resolution reconsidered, and it was laid on the table.

A member from New Jersey hoped that the McClintock case would be brought fairly and squarely before the Association, and that gentlemen would be made to "face the music." It was useless to cloak it, or to attempt to dodge the responsibility.

Dr. Beck, of Indiana, moved an indefinite postponement of the whole subject.

Other gentlemen rose to speak, but the president decided that a motion to postpone was not debatable.

Dr. Jewell rose to a point of order, and protested against being "gagged." (The president here reversed his decision.) Dr. Jewell said that the action of the day previous was regretted, and that gentlemen had acted hastily. Many, who at first sight voted to accept the apologies, now regretted having done so.

Dr. Hamm, of Philadelphia, explained the action of the Philadelphia County Medical Society, and began to read a remonstrance from it, which he desired to incorporate into his speech.

Dr. Biddle objected to the reading of this remonstrance, as a violation of plighted faith.

It was here moved and decided that the Association go into "committee of the whole," and Dr. Edwards, of Ohio, was called to the chair.

A member hoped that there would be no rules of order except what the chair would prescribe.

*The Chair.* "I will prescribe enough." (Laughter.)

Another member inquired if it would be proper to discuss the remonstrance?

*The Chair.* "A gentleman who has the floor can discuss anything on the face of the earth." (Laughter and applause.)

The remonstrance was then read. It was a long document, giving a detailed account of the recommendation by Dr. Reese of Dr. McClintock for a position in Blockley Hospital, after the last-named gentleman had been guilty of selling quack nostrums, and had thus committed an offence against the ethics of the profession.

Dr. Humphries, of Indiana, moved that each member of the committee of the whole be restricted to five minutes, allowing Dr. Reese whatever time he wished to defend himself in.

Dr. Phelps showed that a ten-minutes rule was now in force. Dr. Cox moved, as an amendment, to make the time fifteen minutes; which amendment was lost, and the original motion of Dr. Humphries was then carried.

Dr. Reese then ascended the platform, and made a statement of his position from the commencement of the controversy. He considered his apology of the day previous a satisfactory one, but was willing to make it more so if it was objected to. He had not brought the subject before the Association, but had been given to understand that if he made the apology which he had made the remonstrance would not be offered. During his remarks there was a demand for the reading of the apology; which was read, as follows:

*To the Officers and Members of the American Medical Association:* The undersigned, one of the vice presidents of the American Medical Association, having, during the interval since our last annual meeting, certified to the professional fitness for the charge of the Blockley Hospital, at Philadelphia, of an individual who had been expelled from this body for a violation of our code of ethics, after consultation with the other officers, and yielding to the advice of other personal friends, desires to say to the Association now assembled—

1st. That, in gi[illegible]ing said certificate, he was prompted solely by motives of sympathy and humanity to a fallen brother, who had been a personal friend prior to his offence; and that he did not realize, acting under the impulse of the moment, that his individual act could be construed by the profession as indicating hostility to his brethren.

2d. That while his own mind is clear that his certificate contained only the truth, and that, under his peculiar relations to the party concerned, he could not withhold his certificate, of medical qualification, consistent with conscience and duty, yet he is ready to concede that he had no abstract right to relieve the party from the censure of the Association until this body had restored him to his fellowship.

3d. That, so far from intending any disrespect to the Association, or to its act of discipline, the undersigned had publicly sustained and defended both. He therefore disclaims the inference from his certificate that he intended to recommend to a high professional office a man whom the Association had excluded, and thereby nullify the action of this body.

And, finally, with these statements and disclaimers, the undersigned, while retaining his own opinion of the rectitude of his motives, and of his duty, under the peculiar circumstances of the case, is nevertheless prepared to defer to the judgment of those whom he knows to be his friends, that he erred in doing what he had no right to do, in view of his official position in the Association, and is hence ealled upon to offer this explanation and apology to his brethren.

(Signed) David M. Reese.

It was moved to refer the apology and remarks of Dr. Reese to a special committee of seven, to report to-morrow morning. Dr. Atlee, of Lancaster, and other gentlemen urged delay.

Dr. Payne, of Virginia, asked permission to relate an anecdote. He was reminded of two old Quakers, one of whom kept a store, while the other practised law—both were members of a temperance society, and it was generally thought that the lawyer did not always keep his pledge. One wet, cold day a negro man went to the Quaker's store, and the good man gave him a drink of brandy. This was brought to the notice of the temperance society, and it was decided that the offender should be severely reprimanded. The lawyer was selected to carry out this sentence, and, taking the store-keeper into the woods, he thus addressed him: "Jeemes, thee should be more circumspect!" (Continued laughter.)

Dr. Condie, of Philadelphia, wished to say that he had offered the resolution in good faith, but he denied that he had made propositions to the gentleman from New York, or that the Philadelphia committee had.

Dr. Bowling, of Tennessee, said that there was no question of veracity. Gentlemen on either side were correct. He had heard of misunderstanding, and of probable difficulty, and had earnestly endeavored to arrange it. He had told Dr. Reese that if he made an apology the remonstrance would not be presented, because he had understood gentlemen from Pennsylvania to say so. But he was now aware that these gentlemen did not in any way pledge the Philadelphia county medical society.

Dr. Condie hoped that a committee would be appointed to give the subject a careful consideration.

Dr. Cox, of Maryland, after complimenting Dr. Reese as an able practitioner and an experienced editor, whose labors have been of great value to the profession and to the country, said that he did not consider the statement full and satisfactory. The offence was not an unpardonable one, but the violation of that code of ethics which is the life of the profession should be properly atoned for. (Applause.) The apology was good enough, but it carried as its sting the mental reservation which Dr. Reese persists in. Nay, in his journal, issued simultaneously with this meeting, and circulated here, he says: "Having done right in certifying to the labors of our quondam friend McClintock, we resented the unmerited censures of our Philadelphia brethren." This completely stultifies the effect of the apology.

Dr. La Roche, of Philadelphia, explained his action and that of the Philadelphia county society in the matter.

Dr. Paine, of Vermont, Dr. Cox, and Dr. Bond made some rather sharp remarks. Dr. Davis, of Massachusetts, thought that Dr. Reese had but to admit that he had done wrong, and ask pardon without any mental reservation.

Dr. Reese said that he had intended to make a satisfactory apology. Such was his earnest wish and desire, and he wished to frankly state that he had no mental reservation, neither did he attempt to conceal anything. He made the statement which had been read without reservation and without evasion. (Applause.)

Dr. Condie expressed his entire satisfaction, as did numerous other gentlemen, several crossing to where Dr. Reese was sitting and shaking hands with him.

The committee of the whole then rose, and the chairman reported to the president that the committee had heard and discussed the apology of Dr. Reese, and that they considered that it was "ample, full, complete, and satirfactory."

On motion, the report of the committee was received and adopted.

The case of Dr. Bryan then came up, when it was suggested that his apology should be in writing, he expressing a willingness to make one as ample as was that of Dr. Reese.

Dr. Reese then drafted an apology, but several gentlemen insisted that he should insert the word "regret." Dr. Reese declined, stating that no gentleman would apologize for that which he did not regret, and that he would never be dictated to by any gentleman, even if the prison-door stood open on his right hand, and the stake was at his left hand.

Dr. Wood (who was greeted with loud applause) stated that he had been with the side which had offered the apology, but he did not consider the apology complete without the insertion of the word "regret."

Drs. Bonner, Clark of New Jersey, Hard of Illinois, Parker of New York, and other gentlemen participated in an exciting debate on the necessity of having the word "regret" inserted.

Dr. Reese added the following sentence, "and regrets that he has incurred the displeasure of his brethren." This was not favorably received.

Dr. Boyle, chairman of the committee of arrangements, here announced that arrangements had been made by which delegates who had purchased tickets on their way to the convention over the following roads could return free by exhibiting their cards of membership; Pennsylvania, Wilmington and Manchester, Illinois Central, Northeastern South Carolina, and Richmond and Petersburg.

The apology of Dr. Reese was again taken up, and discussed with spirit, although there was no manifestation of bad feeling on either side. At length he presented the following:

"The undersigned regrets that he certified to the professional qualifications for Blockley Hospital, Philadelphia, of an expelled member of this body, and hereby offers this apology for his departure from the ethical code."

This was received with loud applause, and, on motion of Dr. White, accepted as an ample and satisfactory apology.

Dr. Bryan submitted a similar apology, which was also accepted, and then the committee adjourned until to-day at nine o'clock, A. M., evidently well pleased that this question was finally disposed of.

*Hospitalities.*—At five o'clock, P. M., the delegates went in omnibuses provided for their use to Georgetown College, by invitation of the faculty. After examining this fine institution, which commands a magnificent view, and visiting its fine library, museum, and apparatus room, the party were hospitably entertained; afrer which they returned to this city. In the evening there were entertainments given them at the residences of Dr. Thomas Miller, 246 F street; Dr. Wm. P. ohnson, 466 Seventh street; and Dr. A. Y. P. Garnett, 465 Ninth street.

THIRD DAY.—Meeting opened at 9½ o'clock. The papers of the Secretary not being present, the reading of the minutes of yesterday was deferred.

On motion of Dr. Foster, of Tenn., the reports and communications of the committees on medical topography and epidemics were referred, without reading, to the committee on publication; an amendment that before reference the call be made upon the state committees for their reports, having been rejected.

Dr. Grant, of N. Y., asked leave to present a complaint against the New York Medical College, but upon information by Dr. Edwards that a committee on ethics would be recommended by the nominating committee, he withdrew his request.

The minutes were then read. Several proposals to amend them were made, and either ruled out of order or withdrawn.

The appointment during the last year of Dr. Geo. Haywood, of Boston, as a delegate to represent the American Medical Association in kindred societies in Europe was announced by Dr. Eve.

The deaths of Drs. Marshall Hall, T. Y. Simmons and David Walton were announced.

The committee to whom was referred the subject of a convention of medical college, reported the following preamble and resolutions, through their chairman, Dr. S. D. Gross:

The committee to whom was referred the resolutions appended to the report of the special committee on medical college, have adopted the following preamble and resolution:

Fully appreciating the value and importance of the resolution under which they were appointed, but a majority of the gentlemen constituting this committee not being authorized by the medical faculties of the several colleges with which we are connected to act as their representatives in this matter, and therefore regarding it quite impossible to secure a convention of delegates in the interim of the meetings of the Association; therefore—

*Resolved*, That we recommend to all the medical colleges entitled to a representation in this body, that they appoint delegates, especially instructed to represent them in a meeting to be held at Louisville on Monday, the day immediately preceding the convention of the American Medical Association for the year 1859, at ten o'clock, at such place as the committee of arrangements shall designate.

The reports of special committees for 1858 coming up as the order of the day—

Dr. J. Foster Jenkins, of New York, read a most interesting report on "Spontaneous Umbilical Hemorrhage of the Newly Born," which was succeeded by the reading of a report of the most important character on the "Influences of Marriages of Consanguinity upon Offsprings," from the pen of Dr. S. M. Bemiss, of Ky.

Dr. J. L. Atlee, from the committee on preparing a stone for presentation to the Washington national monument, report that the stone had been prepared of Vermont marble, with a relievo representation of Hippocrates refusing the presents of the Persian king, Artaxerxes, and the inscription "*Vinat Amor Patria.*" The whole the work of a young native artist, J. Augustus Beck, of Leigh, Pa.

Dr. Palmer read an abstract of the report of Dr. E. Andrews, of Ill., on "The functions of different portions of the cerebellum."

Dr. Campbell read the abstract of a report on the "Nervous concomitants of febrile diseases;" which went to the committee on publication.

Dr. J. Marion Sims, of N. Y., illustrated his report on the "Treatment of the results of obstructed labor" by charts; which caused the lady auditors to vacate the gallery. This was a most interesting report, and was listened to with profound attention, and at its conclusion was greeted with loud applause.

Dr. Mark Stevenson, of N. Y., submitted his report on "The treatment best adapted to each variety of cataract," with the method of operation, place, time, &c.; which he accompanied by the exhibition of illustrative drawings.

The following gentlemen were then recommended as chairmen of special committees, on the following subjects:

On the Microscope—Dr. Holsten, Ohio; Medical Jurisprudence—S. Smith, N. Y.; Quarantine—E. Harris, N. Y.; Surgical Pathology—Jas. R.

Wood, N. Y.; Diseases and Mortality of Boarding Schools—C. P. Mattingly, Ky.; Various Surgical Operations for the Relief of Defective Vision—M. A. Pullen, Mo.; Milk Sickness—E. Murphy, Ind.; Blood Corpuscle—A. Sager, Mich.; Medical Ethics—John Watson, N. Y.; Pons Varoii Medulla Oblongata and Spinal Marrow, their pathology and therapeutics—S. B. Richardson, Ky.

These nominations were adopted.

The committee also recommend the passage of a resolution appointing a committee of nine to wait upon the secretary of the treasury, and request the restoration of Dr. M. P. Bailey as inspector of drugs and medicines for the port of New York.

The matter gave rise to a debate, in which Dr. Edwards defended the action of the committee; he said that Dr. Bailey was, in his opinion, the best man in the world for such a post; and he gave a history of the action which secured the passage of the law creating the office. He said that the present incumbent was totally unqualified. Unless such action was had, Drs. Chaffee and Fitch had assured that efforts would be made to repeal the law. It was better to leave to the judgment of each physician the value of important drugs than to allow him to lean on a broken reed.

The subject was debated by Doctors Parker, Wilcox, Wood, Cox, and others. It was strongly objected to the resolution that it savored of politics; but its advocates insisted that it rose above political differences, as it was a question of life and death.

Dr. Cox, of Md., offered a resolution, which was passed, declaring that the appointment of U. S. inspectors of drugs should be msde for moral and scientific qualifications, and not for political bias.

A motion to lay the resolution of the committee on nominations upon the table was lost—ayes 49, noes 64, and the resolution passed—ayes 79, noes 64.

The voluntary essays were, on motion, referred to the committee on publication.

Dr. Edwards was appointed chairman of the committee to apply to the Secretary of the Treasury.

Charges were brought against the New York Medical College by the Newark, N. J., medical association for granting to a quack their diploma.

Similar charges were entered in other cases and referred to the committee on ethics.

Dr. Sutton, of Ky., moved to appoint a committee of three to report a system of uniform registration of births and deaths.

A committee was appointed to urge on the next census bureau certain features of improvement in the manner of taking the census. This committee consists of Drs. Miller, Antisell and Garnett, of Washington city.

A resolution for an interchange of transactions of state and county societies was adopted.

Dr. Boyle, of the committee of arrangements, presented the names of Professor Swallow and Professor Mittag, as "members by invitation," and they were elected.

An invitation from Professor Bache to visit the Coast Survey bureaus, on Capitol Hill, was accepted, and a vote of thanks for the courtesy was passed.

Resolutions of respect for the memory of several physicians who died since the last meeting of the Association passed.

On motion of Dr. Phelps, the following resolutions were passed unanimously:

*Resolved*, That the thanks of this Association are eminently due to the Regents and Professor Henry, of the Smithsonian Institute, for the ample and convenient accommodation afforded for the transaction of business.

*Resolved*, That the committee of arrangements are entitled to our praise and highest appreciation of their exertions to promote the comfort of the members and the best interests of the Association.

*Resolved*, That to the physicians of Washington and Georgetown and the faculty of Georgetown College we accord the homage of our sincerest thanks for their elegant hospitalities extended to the members from abroad, by which the pleasure of their sojourn here has been so greatly enhanced.

*Resolved*, That we feel assured that the impressions on the tablet of memory received here, in our national metropolis, in this the first year of the second decade of the Association, will long remain an evidence of the urbane attentions received not only from the chief magistrate and other public functionaries of our glorious Union, but of private citizens and the community at large.

*Resolved*, That the manifestations of union of heart and purpose in the action of this session inaugurate a new era, and call for devout acknowledgment to Divine Providence, and presage, as we trust, not only a bright future for the Association, but also as contributing to the perpetuity and prosperity of our great national confederation.

Dr. Duhamel, of Washington, moved that a committee be appointed to report upon the "National Hotel disease."

Opposed by Drs. Foster and Boyle, and lost.

Dr. Peter Parker, late commissioner to China, was invited to the stand, and gave an interesting statement of some medical operations in China.

A motion to reconsider the vote appointing a committee to ask the reappointment of Dr. Bailey as inspector of drugs for the port of New York was made by Dr. Dunbar.

A discussion followed, participated in by Drs. Payne, Tyler, Morgan, Palmer, Watson, Burns, and McNulty. The motion was reconsidered—ayes 51, noes 32—and the Association took a recess until five o'clock.

*Evening Session.*—At five o'clock the Association reassembled.

Divers amendments to the constitution of the Association, proposed last year, came up for consideration, and were rejeeted.

The secretary was directed to collect all the by-laws, and have them printed in the next volume.

Various additional votes of thanks were passed, and the Association adjourned *sine die.*

---

*A Nice Thing for Invalids.*—We have always held the opinion, that anything which was good for the well was deserving of solemn consideration, and *a fortiori*, it is evident that we should look with much more respect npon anything which may contribute to the comfort of the invalid, We have lately seen at the establishment of Mr. Victor L. Tiphaine, a dealer in foreign wines, sauces, &c., an individual to whom the profession of Buffalo are especially indebted for the circulation of most palatable and indigestable French *patés* and delicacies generally, some very curious little bottles of excellent cabinet champaigne, holding but half a pint. This seems to us to be just the thing for sick persons, to whom we may prescribe the use of this wine, where only a small quantity is to be taken at a time, and it is desirable to retain the effervescing quality.

We heard that champaigne was occasionally put up in this form, and it immediately occurred to us as very convenient for invalids; accordingly, at our suggestion, Mr. Tiphaine procured a quantity, and we understand that it is not more expensive than the wine in quarts or pints. Truly, luxury keeps pace with the other improvements of the day.

