
JOURNAL INFORMATION
==============================
NLM Title Abbreviation: Buffalo Med J Mon Rev Med Surg Sci
NLM Journal ID: 0004276

The Buffalo Medical Journal and Monthly Review of Medical and Surgical Science

Publisher: Biomedical Journal Digitization Project

ARTICLE INFORMATION
==============================
PMCID: PMC8936069
PMID: 35376955
Article version: 1
Subjects: Editorial Department

Corrections

Print publication date: 1858 Aug
Volume: 14
Issue: 3
First page: 192
Last page: 192
License: This work is free of known copyright restrictions. For more information, please see PMC Back Issue Digitization.
License URL: https://creativecommons.org/publicdomain/mark/1.0/

Erratum for: The Eleventh Annual Meeting of the American Medical Association 14 1 6 1858 46 60 The Buffalo Medical Journal and Monthly Review of Medical and Surgical Science PMC8687887 35376405

==============================

---

*The Ohio Medical and Surgical Journal.*—We notice that the July No. of this valuable journal appears with another name on the cover, Dr. Dawson having associated with him Prof. J. W. Hamilton, Prof. of Surgery in the Starling Medical College.

---

*Medical Society of South-Western New York.*—This society will hold its next meeting at the Jamestown House, Jamestown, on the 4th inst. A good address may be expected from Dr. Hazeltine, and a good time generally.

---

*Not too Late.*—We have yet a few spare copies of Vol. XIII, which we offer to any one gratis, who will send us his name and $2.50. We are not quite exhausted, but soon it will be too late to take advantage of this offer.

---

*What may Happen.*—We are afraid that we shall have to dun in our next number. It will be only dire necessity which will drive us to the alternative of asking for our due, therefore we beg those indebted to us to communicate with Mr. E. R. Jewett, Buffalo, and spare us the humiliation.
